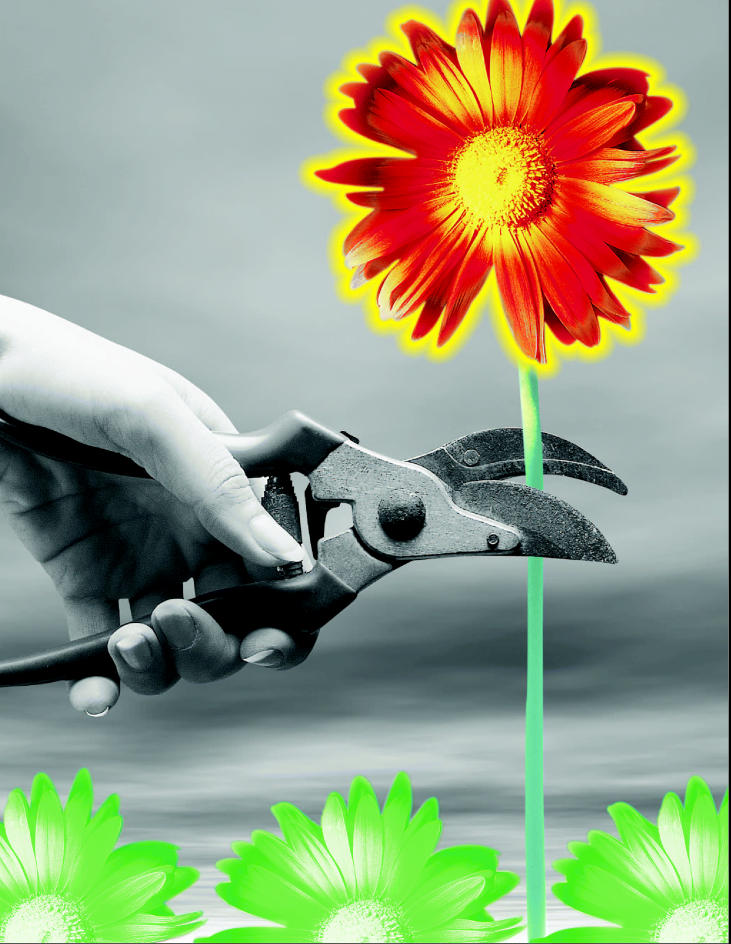# Botanical Supplements: Weeding Out the Health Risks

**DOI:** 10.1289/ehp.112-a750

**Published:** 2004-09

**Authors:** David A. Taylor

For the past decade, the U.S. medical establishment has been adjusting to the rising popularity of herbal remedies and other dietary supplements. The 1994 Dietary Supplement Health and Education Act (DSHEA) created a new regulatory approach for products that included herbal products, vitamins, and minerals. Intended to streamline the entry of lower-risk products to the marketplace, DSHEA has since become viewed by some as having unleashed a deluge of relatively unregulated pharmacologically active products onto unwary consumers. At the same time, reports have mounted describing products of variable quality and manufacturers who lack accountability for their claims. Today, regulatory agencies are ramping up their efforts to ensure the safety of botanical supplements.

## The Last Straw

Under DSHEA, herbal products, vitamins, and minerals were to be regulated as foods, and were not subject to the rigors of drug or food additive approval. Supplements that had been on the market prior to 1994 were presumed to be safe. They could be marketed without further testing or approval from the Food and Drug Administration (FDA), even if they were used in new combinations. [For more on DSHEA, see “Herbal Medicine at a Crossroads,” *EHP* 104:924–928 (1996).]

According to the FDA, the number of supplement products on the market grew more than sevenfold since DSHEA’s 1994 passage, to about 29,000 in 2003. Many manufacturers entered the industry with little investment and easy access to a growing market, and were not careful in processing or labeling their products. Even under the best conditions, quality control is tough for herbal supplements because they start from plants containing many chemical compounds, and constituent concentrations vary from batch to batch. And unlike regulated pharmaceuticals, the active ingredients for botanical medicines and dietary supplements are not well-characterized or in some cases even known.

Consumer concerns about quality cropped up in the late 1990s, as many companies rushed to bring herbal products to mainstream markets. Consumers grew confused by the flood of new brands. Widespread newspaper reports of deaths and other serious adverse reactions, scandals over product labels that misrepresented ingredient content, the discovery of contaminants such as heavy metals, and mixed results in efficacy trials further dampened public enthusiasm. Supplement sales have plateaued since. “Consumers seem to be more skeptical than they were in 1998,” says Floyd Leaders, CEO of Botanical Enterprises, a company that develops natural products.

Nevertheless, botanical supplements were still popular in February 2003, when a pitcher for the Baltimore Orioles major league baseball team died as a result of taking a supplement containing ephedra. Until then, ephedra (also known as *ma huang*) was one of the most popular products for losing weight and enhancing athletic performance, although for years reports of adverse reactions had signaled potential health risks.

Weeks after the baseball player’s death, the FDA proposed a set of mandatory “good manufacturing practices” (GMPs) for dietary supplements to ensure more reliable quality. These resembled practices enacted for over-the-counter drugs, including guidelines for regulating temperature, sanitation, and equipment maintenance. By law, the supplement GMPs followed requirements for food GMPs rather than those for drugs. The proposed GMPs were enacted early this year, with phased-in requirement depending on the size of the manufacturer (large manufacturers must comply within one year, medium-sized manufacturers within two years, and small manufacturers within three years).

Articles in the 26 March and 17 September 2003 issues of *JAMA* raised concerns about ephedra’s safety, drug interactions caused by other supplements, and the rise in unscrupulous advertisements for supplements on the Internet, keeping up the call for regulatory action. At the end of 2003, the FDA finally announced a ban of dietary supplements containing ephedra, which took effect in April 2004. Manufacturers were warned that other risky supplements could be next.

Shortly thereafter, to improve oversight specifically of botanicals used in prescription drugs, the FDA issued a new guidance document, a move that industry watchers found encouraging. The guidance gives incentives for companies to take products through the clinical trials that can lead to stronger claims: if a botanical product is legally marketed in the United States with no known toxic effects, the manufacturer can delay certain preclinical trials on toxicity and move more quickly into the clinical trials phase to determine efficacy. Without gaining premarket approval to sell the product as a drug, a supplement maker can make claims about the product’s effect on the body (so-called structure–function claims, such as “calcium builds strong bones”), but they cannot claim to treat disease (for instance, “calcium reduces the risk of osteoporosis”).

Leaders welcomes the new FDA guidance, which he says puts the agency “two or three years ahead of the industry” in terms of understanding that public perception of an industry’s reliability is critical to product acceptance. What many in the industry don’t yet fully comprehend, says Leaders, is that the new guidance streamlines the process by which manufacturers can gain exclusive claims of benefit. It also gives the FDA a better basis for determining a product’s safety.

The FDA’s pursuit of ephedra brought a shift in the government’s approach to making its case for regulatory action, according to Ilene Ringel Heller, senior staff attorney for the Center for Science in the Public Interest (CSPI), a consumer advocacy group based in Washington, D.C. “What took FDA so long in the case of ephedra is that FDA has the burden of proving that a supplement poses a significant or unreasonable risk before it can take action [to restrict its use],” she says—an extremely difficult case to make in the absence of adverse event reports. “Eventually, FDA decided . . . to do a risk–benefit analysis and decide whether it poses an unreasonable risk. And that’s how ephedra got banned. But it’s not certain that this will be upheld in court.” Furthermore, points out George Lucier, an advisor to the National Toxicology Program (NTP), good risk–benefit analysis is simply not possible without good data on both efficacy and toxicity.

## A New Crop of Research

The framework for federal research on botanical products has evolved to help produce those sorely needed data. The NIH Office of Dietary Supplements (ODS), created by DSHEA, has grown from a $5 million budget in 1999 to one more than five times that size in 2004. Yet according to ODS director Paul Coates, the office’s new five-year strategic plan does not mark a dramatic shift in direction. The new plan has major strategic goals very similar to the ones in the first plan. The difference, says Coates, is one of emphasis: the ODS will continue to support research to improve analytical methods and enhance understanding of the mechanisms by which popular herbal supplements act, and plans to assess the role of dietary supplements in reducing the risk of chronic disease.

In its work, the ODS collaborates with most of the NIH institutes and centers, including the National Center for Complementary and Alternative Medicine (NCCAM), the NIEHS, and the John E. Fogarty International Center for Advanced Studies in the Health Sciences. Referring to NCCAM, Coates says, “We have areas of natural complementarity, given that a great many dietary supplements have been used in traditional healing environments.”

NCCAM’s history parallels that of the ODS: established by Congress in 1998, the center quickly developed a program of research trials that included studies of herbal products used in traditional medical systems such as Indian Ayurvedic medicine and Chinese herbal medicine. NCCAM also established an Office of Clinical and Regulatory Affairs.

Jonathan Berman, director of that office, says the center studies dietary supplements according to a drug model to see if they are safe and effective, and to determine dosage. Throughout its existence, while exploring various alternative therapies, NCCAM has approached dietary supplements with the aim of steering as many as possible toward the FDA’s drug approval process and its benefits of premarket approval and more accountable manufacturing. Berman says NCCAM leaders believe the new FDA guidelines for botanicals used in prescription drugs will lead to safer products all around.

Research on botanical dietary supplements is also being conducted by other branches of the NIH. In recent years, the ODS, NCCAM, and the NIEHS joined forces to create six new research centers devoted to such studies. These university-based centers meet annually to share progress and compare notes, according to Diane Birt, director of the Center for Research on Dietary Botanical Supplements at Iowa State University. Other centers are located at the University of California, Los Angeles (Center for Dietary Supplements Research: Botanicals), the University of Illinois at Chicago (Center for Botanical Dietary Supplement Research in Women’s Health), the University of Missouri–Columbia (Center for Phytonutrient and Phytochemical Studies), Purdue University and the University of Alabama at Birmingham (Botanicals Research Center for Age Related Diseases), and the University of Arizona (Center for Phytomedicine Research).

Researchers at the NIEHS and the NTP also are conducting studies on the safety of compounds found in dietary supplements. Since 1998, the NTP has performed literature reviews for 41 candidate substances, with studies actually conducted on about 20. Among the substances studied were the alkaloids in comfrey (*Symphytum officinale*), an herb used by the ancient Greeks to stop bleeding and heal wounds. Comfrey, however, contains pyrrolizidine alkaloids, which are known to cause liver cancer (in 1993 the FDA cited these effects in its report *Unsubstantiated Claims and Documented Health Hazards in the Dietary Supplement Marketplace*). The NTP quantified the alkaloid content of comfrey samples and relayed the results to the FDA, and in July 2001 the FDA advised manufacturers to take comfrey products off the market. “Although the FDA cannot require toxicological data for herbal products, they can take regulatory action if data indicative of risk become available. So studies like the NTP studies are important for addressing public health concerns,” says Lucier.

Besides preclinical studies, the ODS supports efforts to standardize methods for assessing supplements, as Congress mandated in 2002. This mandate answered a need voiced by the industry itself, according to Michael McGuffin, president of the American Herbal Products Association (AHPA), a trade association based in Silver Spring, Maryland. The industry welcomes standardized methods, McGuffin says, so that the same analytical methods are used by producers, the media, and the FDA alike. This can avert conflicting safety and efficacy reports that leave consumers baffled.

Some experts maintain that even the big NCCAM-supported clinical trials are not as definitive as they should be. One such major study, focusing on St. John’s wort (*Hypericum perforatum*) and published 10 April 2002 in *JAMA*, found the herb to be “no better than placebo for the treatment of major mental depression,” says Berman. Observers inside and outside the industry were dismayed perhaps less by the findings than by how they were reported. “There appeared to be bias in the reporting,” Leaders says—what the medical journal didn’t note was the authors’ finding that the currently accepted treatment for depression likewise performed no better than placebo, thus casting doubt on the entire experiment.

Adds Mark Blumenthal, who directs the nonprofit American Botanical Council of Austin, Texas, “Just as there have been problems with quality control in some aspects of the herbal industry, there are also some serious problems of quality control in the way that the media and the medical journals themselves have reported on the herbals.”

Coates acknowledges the need for clearer public information. “The people who commented on our strategic planning process more than once said that we have to pay more attention to the development of appropriate communications and information tools,” he says, “and we’re planning to do that.”

## Growth Under DSHEA

In recent years, the FDA has reorganized itself to better reckon with the challenges posed by DSHEA. In February 2003, the Office of Nutritional Products, Labeling, and Dietary Supplements created a separate Division of Dietary Supplement Programs. Susan Walker, who heads that division, says it continues to explore relationships with the NIH and its research institutes, including the NTP, and to coordinate with other parts of the FDA, notably the National Center for Toxicological Research.

The FDA and other agencies have become more vigilant in enforcing laws pertaining to supplements. In late 2003 and early this year, the FDA sent 119 warning letters to distributors of supplements and refused entry of over 1,100 imported products, according to a 19 April 2004 agency press release. Enforcement by other agencies has been stepped up too. The Federal Trade Commission, the FDA’s partner in enforcement, cracked down on unscrupulous advertisers of dietary supplements in 2003 as part of an ongoing effort known as Operation Cure-All. The result was 83 warning letters demanding that companies cease making illegal claims on their websites and in their literature.

Manufacturers, too, are finding ways to live with the new policies. In the past, companies complained that there was no incentive for drug research on herbs because they couldn’t patent a natural product. But there are ways around that obstacle, for example by patenting the process for extraction. “The process defines the product,” says Leaders. He gives the example of brewing coffee: Starting from the same coffee beans, you can either brew very strong Turkish coffee or a drink as weak, he says, “as my mother used to make.”

Leaders says one of the two main weaknesses of the 1994 law was that it did not require manufacturers to report adverse reactions to the FDA. The latter point was echoed recently by a review panel of the Institute of Medicine in its April 2004 report *Dietary Supplements: A Framework for Evaluating Safety*, and by consumer groups. “FDA needs authority to require mandatory adverse event reporting,” says Heller. “It’s a major shortcoming that FDA doesn’t have it.”

The FDA does have a voluntary system in place for reporting adverse events—the Center for Food Safety and Applied Nutrition Adverse Event Reporting System is a computerized database of records submitted by consumers, health care providers, and industry. Yet despite some revamping of the system, Heller says the fact remains that reporting to this system is, by virtue of being voluntary, largely ineffective. Indeed, the April 2001 Department of Health and Human Services report *Adverse Event Reporting for Dietary Supplements: An Inadequate Safety Valve* cites an unnamed FDA-commissioned report as finding that the FDA receives reports of less than 1% of all adverse events associated with dietary supplements.

Other nations, too, are looking at issues related to botanical supplement safety. Both the European Union and Canada have added a new “traditional medicine” category for products that have a history of use in the literature without adverse reactions. Like the United States, Europe is reviewing how to assess the risks and benefits of botanical dietary supplements. An expert group of the European branch of the International Life Sciences Institute (ILSI Europe), a Brussels-based nonprofit foundation that is funded primarily by industry members, published a paper in the December 2003 issue of *Food and Chemical Toxicology* offering new guidance on how to assess the safety of botanicals. The authors stated that “ultimate safety in use depends on the establishment of an adequate safety margin.” This margin is the ratio between the dose demonstrated to be safe from research and the dose actually consumed, explains Nico van Belzen, executive director of ILSI Europe.

Defining what size margin is adequate, however, is a thorny proposition and requires a case-by-case determination, admits van Belzen. ILSI Europe suggests a decision tree approach to the evaluation process, and has created a model for risk–benefit analysis of micronutrients that can provide a tool to help officials weigh the risk of deficiency (making the dosage too low for users to experience a product’s benefit) against the risk of toxicity (exceeding the tolerable upper limit). That model will be published in an upcoming issue of *Food and Chemical Toxicology*.

In the shadow of the ephedra mêlée, the system for balancing the potential risks and benefits of botanical dietary supplements clearly is still taking shape. With better understanding of herbal compounds and improved regulation of the claims made on their behalf, however, manufacturers and the public alike could reap a rich harvest.

## Figures and Tables

**Figure:**